# Diversity of Antimicrobial Peptides in Three Partially Sympatric Frog Species in Northeast Asia and Implications for Evolution

**DOI:** 10.3390/genes11020158

**Published:** 2020-02-01

**Authors:** Qing Wang, Rui Xia, Jing Jing Ji, Qian Zhu, Xiao Ping Li, Yue Ma, Yan Chun Xu

**Affiliations:** 1 Department of Physiology, College of Wildlife and Protected Area, Northeast Forestry University, Harbin 150040, China; wangqingtianxie@163.com (Q.W.); xiarui_19870203@163.com (R.X.); jinglove19860000@126.com (J.J.J.); zhuqian@yxvzb.net (Q.Z.); bingxuaner123@163.com (X.P.L.); dornmark@163.com (Y.M.); 2 Department of Ecology, School of Life Sciences, Lanzhou University, Lanzhou 730000, China; 3 BGI-Shenzhen, Shenzhen 518083, China; 4 Beijing E-young Technology Company Limited, Beijing 100021, China; 5 State Forestry and Grassland Administration Detecting Centre of Wildlife, Harbin 150040, China; 6 State Forestry and Grassland Administration Research Center of Engineering Technology for Wildlife Conservation and Utilization, Harbin 150040, China

**Keywords:** antimicrobial peptides, transcriptome, diversity, frog, adaptive evolution

## Abstract

Antimicrobial peptides (AMPs) are evolutionarily ancient molecules that play an essential role in innate immunity across taxa from invertebrates to vertebrates. The evolution system of AMP system has not been well explained in the literature. In this study, we cloned and sequenced AMP transcriptomes of three frog species, namely *Rana dybowskii*, *Rana amurensis*, and *Pelophylax nigromaculatus*, which are partially sympatric in northeast Asia, but show different habitat preferences. We found that each species contained 7 to 14 families of AMPs and the diversity was higher in species with a large geographic range and greater habitat variation. All AMPs are phylogenetically related but not associated with the speciation process. Most AMP genes were under negative selection. We propose that the diversification and addition of novel functions and improvement of antimicrobial efficiency are facilitated by the expansion of family members and numbers. We also documented significant negative correlation of net charges and numbers of amino acid residues between the propiece and mature peptide segments. This supports the Net Charge Balance Hypothesis. We propose the Cut Point Sliding Hypothesis as a novel diversification mechanism to explain the correlation in lengths of the two segments.

## 1. Introduction

Amphibians live in humid or aquatic environments that support the growth of complex microbial communities, including pathogenic microbes. Unlike higher vertebrates whose bodies are well protected by a thick and hard keratin layer, this class of organisms has only one or two layers of mild keratinocyte cells on the body surface, making the skin highly permeable and vulnerable [1,2]. Such a weak physical barrier cannot provide guaranteed protection from pathogenic attacks. As compensation, amphibians have a powerful antiseptic barrier in the skin that is made up of antimicrobial peptides (AMPs), small cationic and amphipathic peptides highly efficient at destroying microbial pathogens [3,4]. These are synthesized, matured, and stored in granular glands, and dissolved in the mucosal layer from which they attack microbes that invade the skin [5,6].

AMPs are composed of ~10–60 amino acid residues, with a molecular mass of approximately 4000–6000. They occur naturally in all living beings and carry out highly specific biological activities, whose specificity is mainly based on and dependent on their primary sequences and, ultimately, on their conformational structure. They are cationic and amphipathic molecules, hydrophilic at the N end and hydrophobic at the C end. These cationic characteristics and amphipathicity are the key features conferring an antimicrobial capacity on AMPs [7]. AMPs are arranged in an α helix, β folding, and loop structures [8], and are stable in hot and acid environments [9]. They resist bacteria through mechanisms such as membrane interference, inhibition of nucleic acid synthesis, protein synthesis, and enzymatic activity [10]. In addition, many AMPs have antiviral, antifungal, antiprotozoal, and antitumoral capacities [11].

AMPs are highly diverse. Amphibian species might synthesize up to 66,000 different kinds of AMPs [12,13]. They can be sorted into different families according to their structure and physicochemical properties [14]. Variation of amino acid residues of family members often alters the secondary structure and physicochemical properties, and further shifts antimicrobial efficiency and spectrum. It is thus apparent that expansion of family characteristics and boosting of variation may improve defense capability. Gene duplication and focal point mutation are driven by positive selection [15,16]. Consequently, an anuran genome contains several gene families with a variable number of member genes encoding a large batch of AMPs. Meanwhile, regulatory events such as an increased mutation rate and multiple gene loci during expression further facilitate the diversity of the AMP profile [16,17]. An individual anuran may contain 10–20 AMPs of different lengths, net charges, hydrophobicity, structures, and antimicrobial spectra [18,19]. The expression profile can be contingent on environmental context; exposure to varied microbial communities can result in expression of AMP profiles that vary by species and quantity of AMPs [20].

The AMP system is the most important component of innate immunity in amphibians. Its evolution is thus an interesting and important subject. Hypotheses on the mechanism of evolution include positive (diversifying) selection [16], balancing selection [21], hypermutation [22], and domain shuffling or gene conversion among AMP genes [23]. However, these hypotheses have proven inadequate to describe the entire process of evolution of the AMP system. To complete the evolutionary picture, systematic studies are needed at the biochemical (structure-function relationship), cellular (signaling and expression regulation), physiological (immune efficiency), and ecological (relationship between AMP immunity and environmental microbial pressure) levels. Such studies could explain how selection pressure is placed on an AMP profile, how a single specific AMP responds to a type of selection pressure under the influence of regulatory mechanisms, and how its gene evolves under such selection. To achieve this, it essential to provide a full list of expressible AMP genes as the background/potential expression profile. However, most studies have been performed under specific environments that preclude expression of the full range of background expression profiles [16,22].

In this study, we examined three frog species of northeast Asia, viz. Amur brown frog (*Rana amurensis*), Dybowski’s frog (*Rana dybowskii*), and black-spotted frog (*Pelophylax nigromaculatus*). Although their geographic ranges partially overlap, their habitats are different. The range of *R. dybowskii* is limited to northeast Asia, including the Russian Far East, Korean Peninsula, Tsushima Island of Japan, and northeastern China. In contrast, *R. amurensis* is widely distributed in the Palearctic region across Siberia, the Russian Far East, the Korean peninsula, northern and central Mongolia, and northeastern China. *P. nigromaculatus* ranges from the Russian Far East and Turkmenia, northern to south China, the Korean Peninsula, and Japan [24]. *P. nigromaculatus* inhabits a wider variety of habitats than do the other two species. These species can share common habitats but also have discrete habitat preferences where their ranges overlap [25,26,27]. This makes them ideal models to study AMP evolution under variable ecological contexts. This study established the background AMP transcriptomes of the three species by sampling frogs from various habitats, predicted AMPs’ structural and physicochemical characteristics, and documented new findings that highlight novel evolutionary mechanisms. Meanwhile, we added a set of novel AMPs to the growing database for anurans, information that could benefit future biological studies and/or drug development.

## 2. Materials and Methods 

### 2.1. Collection of Skin Tissues

A total of 12 adult frogs of each of our three study species were collected from various habitats in northeast China, including riverbanks (*n* = 3 for each species), paddy fields (*n* = 3 for each species), ponds in woodlands (*n* = 3 for each species), and natural saline-alkaline wetlands (*n* = 3 for *P. nigromaculatus* only because the other two species did not occupy this habitat). Three additional individuals of each species were captured from riverbanks and raised in the lab for 3 weeks in fully aerated fresh water. Water was replaced once each day and the frogs were fed mealworms. Three biological repetitions were evaluated for each habitat. The frogs were killed by pithing the spinal cord and brain. Dorsal skin was removed and immediately frozen in liquid nitrogen for storage prior to study.

### 2.2. Cloning of cDNA of Antimicrobial Peptide (AMP) Genes 

A piece of frozen dorsal skin weighing about 100 mg was ground into powder in liquid nitrogen. Total RNA was extracted using the Trizol method (TRIzol, TaKaRa, Japan) according to the manufacturer’s instructions. After quantification using NanoPhotometer-N50 (Implen, Germany), all RNA extracts of each species were pooled equally. Complementary DNA (cDNA) was synthesized using an RNA PCR Kit (AMV) Ver3.0 (TaKaRa, Japan). Briefly, a universal primer PC: 5′-T23 (C/G/A)-3′ was used to synthesize the first-strand cDNA with input of 500 ng pooled total RNA in a 10 μL system following the instructions provided in the user manual of the kit. The reaction program consisted of 42 °C/30 min, 99 °C/5 min, and 5 °C/5 min.

A total of 10 degenerated forward primers, named PS1~PS10, were designed based on the conservative sequence in the signal peptide region of known anuran AMP genes using Primer-BLAST (NCBI), theoretically amplifying all families of AMPs of anurans. The universal primer PC was used as the reverse primer. All primer sequences are shown in Table 1. PCR amplifications were carried out using reverse transcription products as templates in a 50 μL system containing 10 μL 5× PCR buffer and 0.25 μL TaKaRa Ex Taq HS (5 U/μL) (TaKaRa, Japan), 1 μL (10 μmol/μL) forward primer, and 10 μL template. Thermal cycling program was 94 °C/2min, (94 °C/30s; Ta/30s; 72 °C/30s) for 35 cycles; 72 °C/5 min. PCR products were separated on 1.5% agarose gel. The target fragments were excised and recovered using DNA purification kit (AXYGEN, USA).

PCR products were ligated to the pMD-18T vector and transformed into *Escherichia coli* DN5α competent cells using a pMD-18T connection kit (TaKaRa, Japan). The competent cells were cultured for 12–16 h on LB medium containing 50 μg/mL Ampicillin. Single colonies were picked up and transferred to liquid LB medium for culture for about 2–3 h till OD600 reached 0.5. A sample of the bacteria solution measuring 1 μL was used as template for screening positive clones in the same PCR system as mentioned previously. Positive clones were sequenced using M13+/M13-primers using the BigDye Terminator v3.1 Cycle Sequencing Kit (Thermo Fisher Scientific, USA).

### 2.3. Prediction of Physicochemical Properties and Secondary Structure of Antimicrobial Peptides (AMPs)

For obtained sequences, vector sequence was removed from AMP sequence using the software EditSeq in the DNAstar software package (version 7.1.0, DNASTAR, Madison, Wisconsin, USA). All sequences were aligned and compared using MegAlign software in the same package. A cDNA sequence was determined using identical sequences from at least three different clones. Each cDNA sequence was blasted in the GenBank database (accessed on 20 October 2015). The signal peptide, acidic propiece, and mature peptide region was determined according to the annotation of homogeneous peptides. The amino acid logos of the mature peptides for all three frog species were created through the website WebLogo 3 [28]. Phylogenetic trees were constructed for all AMP cDNA sequences after the untranslated region was cut off. Trees were inferred by Akaike Information Criterion (AIC) for automatic model selection using PhyML-SMS on the Booster website [29]. The stability of phylogenetic trees was tested by bootstrap resampling analysis of 1000 replicates using BOOSTER [30], using only the branches with transfer bootstrap expectation (TBE) higher than 60. The tree was embellished at the EvolView website [31]. With reference to homologous sequences in GenBank, each clade with common characteristics, including length of each region, putative sequence, and secondary structure of matured peptide, was assigned to a family. For novel AMP sequences that had no homologous sequence registered in GenBank, we named them according to the nomenclature guideline [32]. All confirmed AMP gene sequences were deposited to the NCBI database. Meanwhile, DnaSP (version 6.12.03, Universitat de Barcelona, Barcelona, Spain) [33] was used to analyze the nucleotide diversity of the cDNA sequences of each AMP family for the three frog species, expressed as Mean Pi value ± SD.

The cDNA sequence of each AMP was translated into amino acid sequences using EditSeq software. Theoretical molecular weight, isoelectric point, hydrophilicity, and stability were calculated using the tools provided at the online website ExPASy [34]. Secondary structural characteristics and solubility were predicted by means of websites [35,36]. Then, the correlation between the length of acidic propiece and the length of mature peptide, and between the net charge of propiece and mature peptide were statistically analyzed by SPSS (version 19.0, IBM, Armonk, NY, USA)using 266 AMP amino acid sequences.

To test the selection pressure of genes in each AMP family, the ratio of nucleotide non-synonymous mutation rate (d*_N_*) to synonymous mutation rate (d*_S_*), d*_N_*/d*_S_*, usually expressed as omega (ω) was tested using the codemL program in PamL4.9h (version 4.9h, Ziheng Yang, University College London, London, England) under different codon substitution models M0 (one ratio) [37]. Using the same set of frog sequences, we also examined the transition (*t_s_*)/transversion (*t_v_*) values of the propiece and the mature peptide with the program codemL.

## 3. Results

### 3.1. Cloning and Sequence Analysis of Antimicrobial Peptide (AMP) cDNAs

The total RNA extracted from the skin tissue showed two clear bands on 2% agarose gel under UV. The ratio of OD260/OD280 was around 2.0 and OD260/OD230 was greater than 1.8, demonstrating that the quality was high enough to support subsequent experiments. The total RNA of all samples was diluted to about 500 ng/μL with RNase-free water.

A total of 2718 positive clones were sequenced from the three frog species, 915 for *R. amurensis*, 879 for *R. dybowskii*, and 924 for *P. nigromaculatus*. A cDNA sequence was accepted for further analysis only if it possessed characteristics of AMP genes and was identical for at least three clones. In total, we obtained 81 cDNA sequences from *R. amurensis*, 79 sequences from *R. dybowskii*, and 106 sequences from *P. nigromaculatus*.

**Figure 1:**
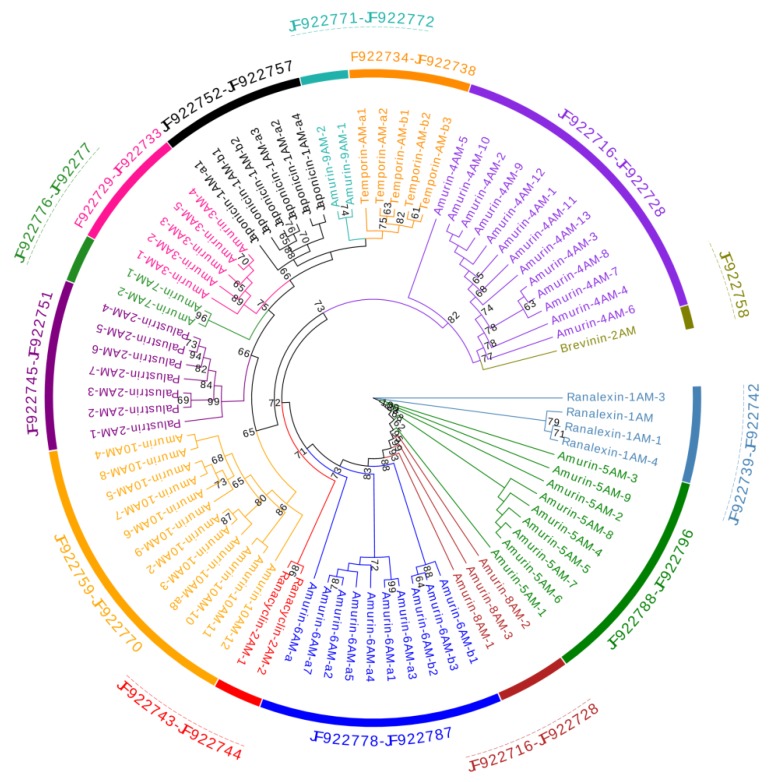
A phylogenetic tree of antimicrobial peptide (AMP) genes identified from *R. amurensis*. The tree was constructed based on the sequence of acidic propiece and mature peptide region. Numbers in the figure are confidence values with bootstrap 1000 replicates; only values ≥60 are shown.

The phylogenetic tree (Figure 1) demonstrated that the 81 AMP cDNA sequences of *R. amurensis* fell into 14 clades. Deduced amino acid sequences of 6 clades were homologous to 6 reported AMP families namely brevinin-2AM, japonicin-1AM, palustrin-2AM, ranacyclin-2AM, ranalexin-1AM, and temporin-AM. We did not find AMP records homologous for the remaining eight clades. Their deduced amino acid sequences were different from each other with unique characters. Therefore, we assigned a family name to each according to the nomenclature guideline: amurin-3AM, amurin-4AM, amurin-5AM, amurin-6AM, amurin-7AM, amurin-8AM, amurin-9AM, amurin-10AM.

**Figure 2:**
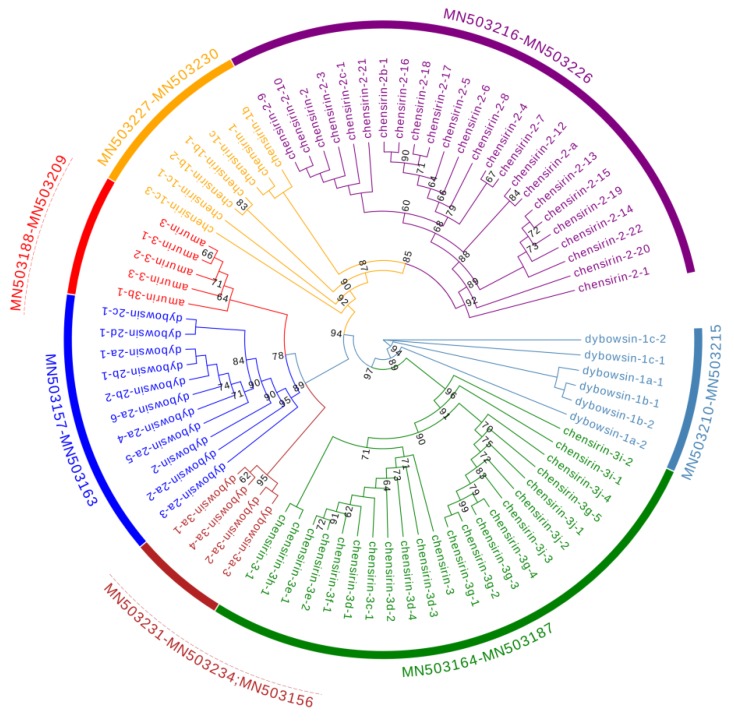
A phylogenetic tree of antimicrobial peptide (AMP) genes identified from *R. dybowskii*. The tree was constructed based on the sequence of acidic propiece and mature peptide region. Numbers in the figure are confidence values with bootstrap 1000 replicates; only values ≥60 are shown.

The 79 AMP cDNA sequences of *R. dybowskii* were clustered into 7 different clades, 4 of which were homologous to 4 AMP families, including chensirin-1, chensirin-2, chensirin-3, and amurin-3. Three clades had not been reported, and were named as dybowsin-1, dybowsin-2, and dybowsin-3 (Figure 2).

**Figure 3:**
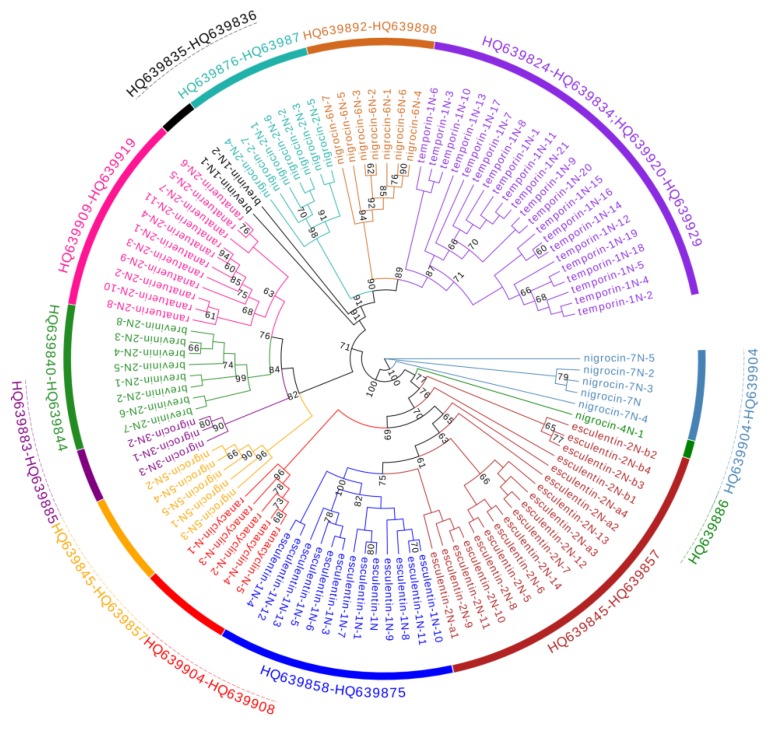
A phylogenetic tree of antimicrobial peptide (AMP)genes identified from *P. nigromaculatus*. Numbers in the figure are confidence values with bootstrap 1000 replicates; only values ≥60 are shown.

The 106 cDNA sequences of *P. nigromaculatus* were clustered to 13 clades (Figure 3). Seven clades corresponded to reported AMP families, namely brevinin-1N, brevinin-2N, esculentin-1, esculentin-2, ranacyclin-N, ranatuerin-2N, and temporin-1. The other six clades were novel AMPs, and were assigned family names, viz, nigrocin-2N, nigrocin-3N, nigrocin-4N, nigrocin-5N, nigrocin-6N, and nigrocin-7N.

All cDNA sequences were deposited to GenBank. The accession numbers were: JF922716-JF922796 for *R. amurensis* cDNAs, MN503156-MN503234 for *R. dybowskii* cDNAs, and HQ639845-HQ639834 for *P. nigromaculatus* cDNAs.

### 3.2. Nucleotide Diversity of Antimicrobial Peptide (AMP) Families

**Figure 4:**
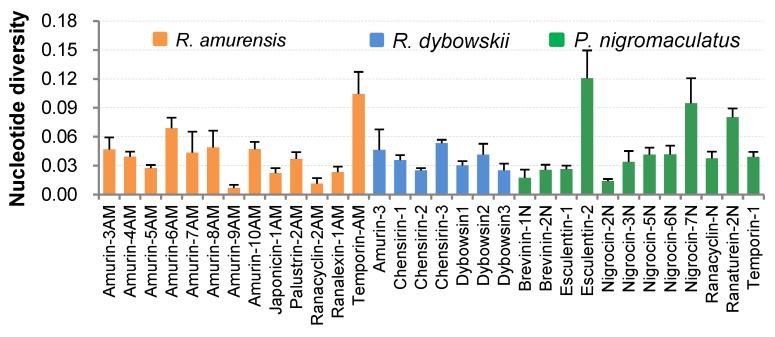
Nucleotide diversity (±SD) of antimicrobial peptide (AMP) families identified from the three anuran species.

Except for nigrocin-4N in *P. nigromaculatus* and brevinin-2AM in *R. amurensis*, which had only one cDNA sequence each, nucleotide diversity (π) of the remaining 32 AMP families was analyzed (Figure 4). The average nucleotide diversity of all AMPs of *R. amurensis* ranged from 0.007 ± 0.003 to 0.104 ± 0.023, averaging 0.040 ± 0.010. The highest diversity was seen in the family temporin-AM (average 0.104 ± 0.0023), and the least in family amurin-9AM (0.007 ± 0.003). The nucleotide diversity of AMP families varied little even in *R. dybowskii*, for which π values ranged between 0.025 ± 0.002 and 0.053 ± 0.003, averaging 0.104 ± 0.023. For *P. nigromaculatus*, π values of 9 of 12 AMP families also varied little, ranging from 0.014 ± 0.002 to 0.042 ± 0.009 with a mean 0.031 ± 0.007. However, three families namely esculentin-2, nigrocin-7N, and ranaturein-2N had high variation and set the upper limit at 0.121 ± 0.029. The average π value of the three families was 0.099 ± 0.021.

### 3.3. Divergence Pattern of Antimicrobial Peptides (AMPs) in Three Species

**Figure 5:**
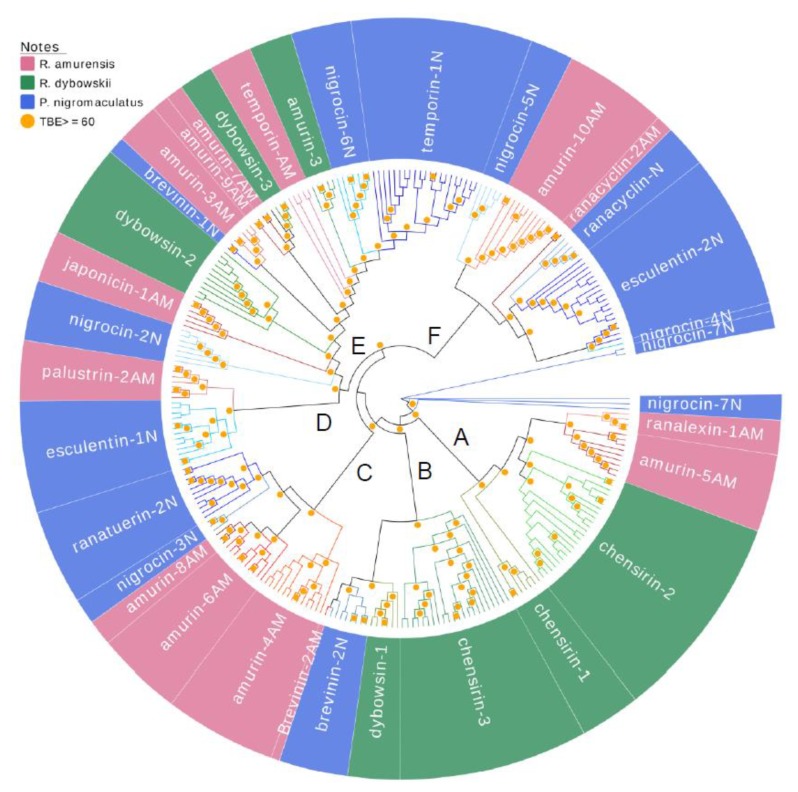
A phylogenetic tree of all antimicrobial peptide (AMP) genes identified from three anuran species, namely *R. amurensis* (pink), *R. dybowskii* (green) and *P. nigromaculatus* (blue). Orange dots in the figure are confidence values with transfer bootstrap expectation (TBE) 1000 replicates; only values ≥60 are shown.

A comprehensive phylogenetic tree was constructed for all 266 AMP cDNAs cloned from the three frog species using the Neighbor-joining method (Figure 5). Similar to the cluster patterns in Figure 1, Figure 2 and Figure 3, the cDNAs of each family were perfectly clustered, and most branching was strongly supported by bootstrapping, shown as transfer bootstrap expectation (TBE) of 1000 replicates greater than 60. All main clades consisted of several families and showed both intra-specific and inter-specific diversification. For instance, clade A first diverged as a branch of family chensrin-1, then diverged into two clades, viz. the chensrin-2 clade and the ranalexin-1-amurin-5AM clade. The former clade was private to *R. dybowskii* and the later clade was private to *R. amurensis* but diverged intra-specifically into two families. Another example is clade B that split into two clades; one is the family chensrin-3 that diverged within the species *R. dybowskii*, and the other further diverged into two clades, dybowsin-1 that is private to *R. dybowskii* and brevinin-2N. However, a brevinine-2N gene further diverged inter-specifically to be brevinin-2AM that is possessed by *R. amurensis*. The most complex clade showing intra- and inter-specific divergence was clade E, in which all families presented deep sequence diversification within species and clades split into families belonging to different species.

### 3.4. Prediction of Physicochemical Properties and Secondary Structure of Antimicrobial Peptides (AMPs)

**Figure 6:**
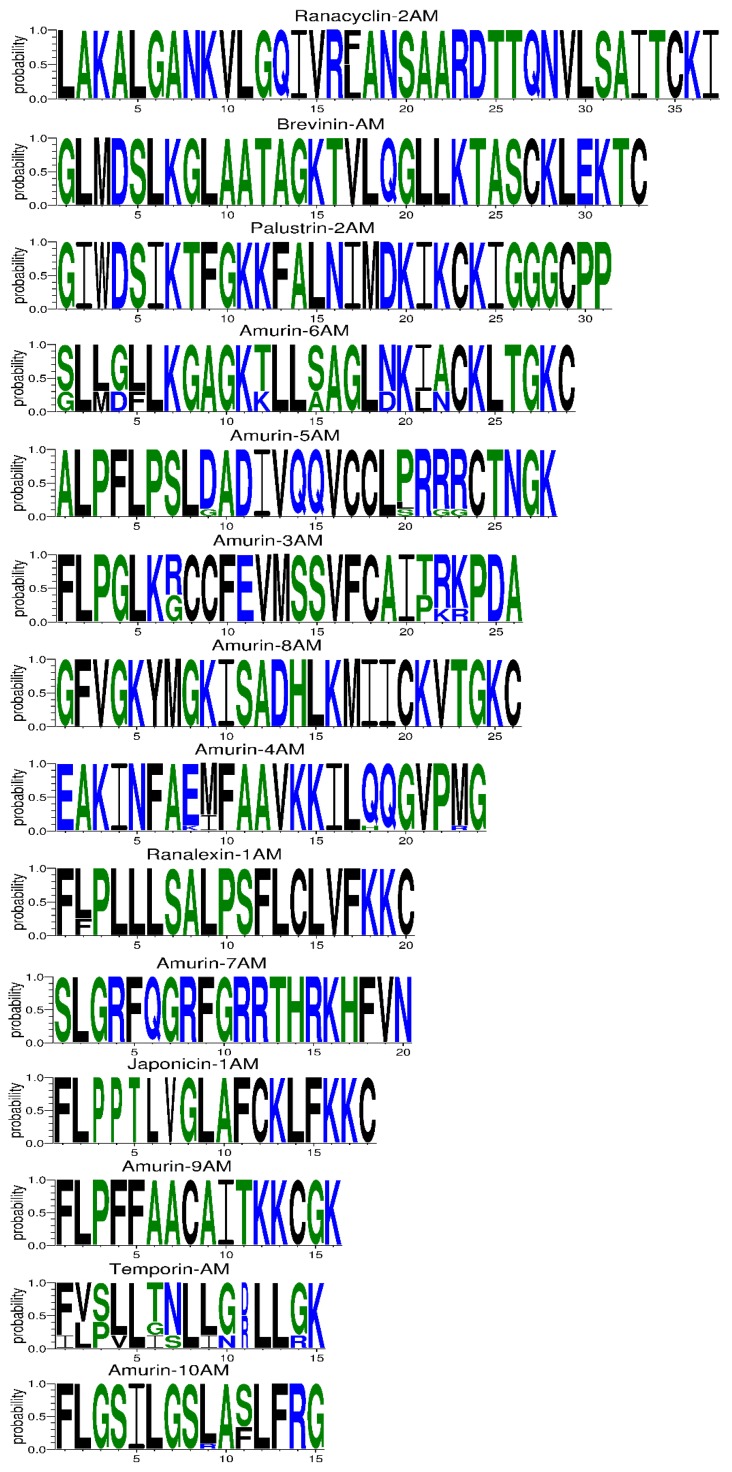
The primary structure of mature peptides of 14 AMP families from *R. amurensis*. The blue letters represent hydrophilic residues, including Arginine (R), Lysine (K), Aspartic acid (D), Glutamic acid (E), Asparagine (N), Glutamine (Q); the green letters represent neutral residues, namely Serine (S), Glycine (G), Histidine (H), Threonine (T), Alanine (A), and Proline (P); the black letters represent hydrophobic residues, including Tyrosine (Y), Valine (V), Leucine (L), Isoleucine (I), Phenylalanine (F), Tryptophane (W), Methionine (M), and Cysteine (C).

**Figure 7:**
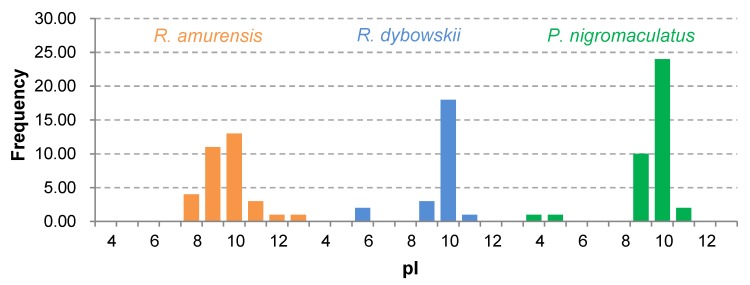
Frequency distribution of theoretical isoelectric points (pI) of antimicrobial peptides (AMPs) isolated from the three anuran species.

**Figure 8:**
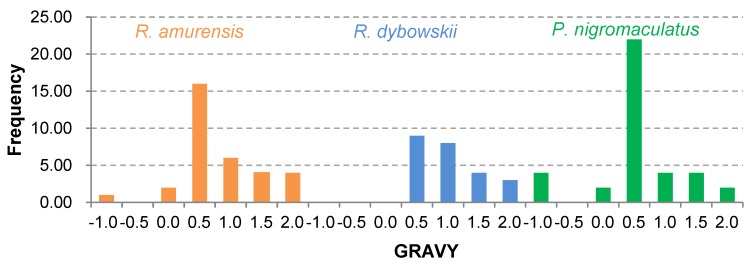
Frequency distribution of hydrophobicity predicted for all antimicrobial peptides (AMPs) isolated from the three anuran species.

**Figure 9:**
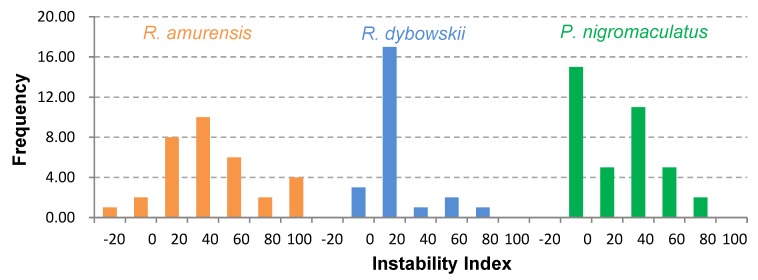
Frequency distribution of Instability index (II) values of all antimicrobial peptides (AMPs) isolated from the three anuran species.

The 81 AMP cDNA sequences isolated from *R. amurensis* encoded 33 mature peptides belonging to 14 families (Figure 6). They were rich in Leucine (L) and Glycine (G). Family amurin-3AM and amurin-4AM were also rich in hydrophobic amino acid residues Phenylalanine (F) and Alanine (A). The molecular weight of these mature peptides ranged from 1487.8 to 3857.5. The theoretical isoelectric point (pI) values ranged from 7.94 to 12.60, averaged 9.34 ± 1.06, suggesting that they are alkaline AMPs (Figure 7). All peptides showed hydrophobicity, except for amurin-7AM that showed strong hydrophilicity (GRAVY = −1.31) (Figure 8). The instability index varied widely (II = −23.21 to 87.83). Family amurin-5AM, amurin-3AM, and amurin-9AM had high instability index (74.29, 77.14, and 55.61, respectively), suggesting low stability. In contrast, all other families were stable with instability indices ranging from −23.21 to 42.19 (Figure 9). Prediction of secondary structure showed that 66.7% of mature peptides were in allα conformation, others were mixed (Table A1).

**Figure 10:**
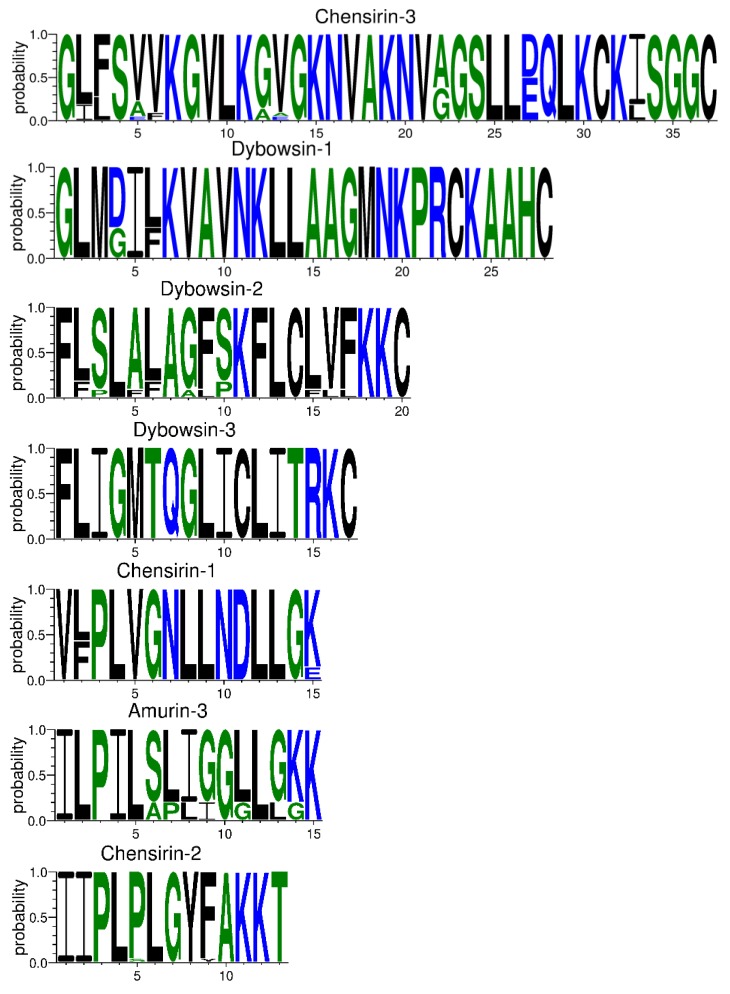
The primary structure of mature peptides of 7 antimicrobial peptide (AMP) families from *R. dybowskii*. The blue letters represent hydrophilic residues, including Arginine (R), Lysine (K), Aspartic acid (D), Glutamic acid (E), Asparagine (N), Glutamine (Q); the green letters represent neutral residues namely Serine (S), Glycine (G), Histidine (H), Threonine (T), Alanine (A), and Proline (P); the black letters represent hydrophobic residues, including Tyrosine (Y), Valine (V), Leucine (L), Isoleucine (I), Phenylalanine (F), Tryptophane (W), Methionine (M), and Cysteine (C). 63.6% sequences (14 out of 22) of chensirin-3 had deletion of a short fragment spanning site 19 to 22; 80.0% sequences (1 out of 5) of amurin-3 had an amino acid deletion at site 15.

The 79 AMP cDNA sequences from *R. dybowskii* encoded 24 mature peptides of 7 families (Figure 10). All families were rich in Leucine (L), taking the total number of residues up to 40%. Except for family dybowsin-3 and chensirin-2, all families were also rich in Glycine (G). Family chensirin-3, dybowsin-1, dybowsin-2, and dybowsin-3 were enriched in hydrophobic amino acid residues, such as Valine (V), Alanine (A), Isoleucin (I), and Phenylalanine (F). The molecular weight ranged from 1406.8 to 3732.4. The pI ranged from 8.75 to 10.07, except for the family chensirin-1, whose pI was as low as 5.81 (Figure 7). All AMPs were hydrophobic with a compact pattern of pI values (Figure 8). Except for the family amurin-3 (53.31, 63.26) and chensirin-2 (43.01) that were predicted to be unstable, most of the AMPs showed high stability (Figure 9). The family chensirin-2 was in free curly structure, namely mixed, and all other AMPs were in all-α conformation (Table A2).

**Figure 11:**
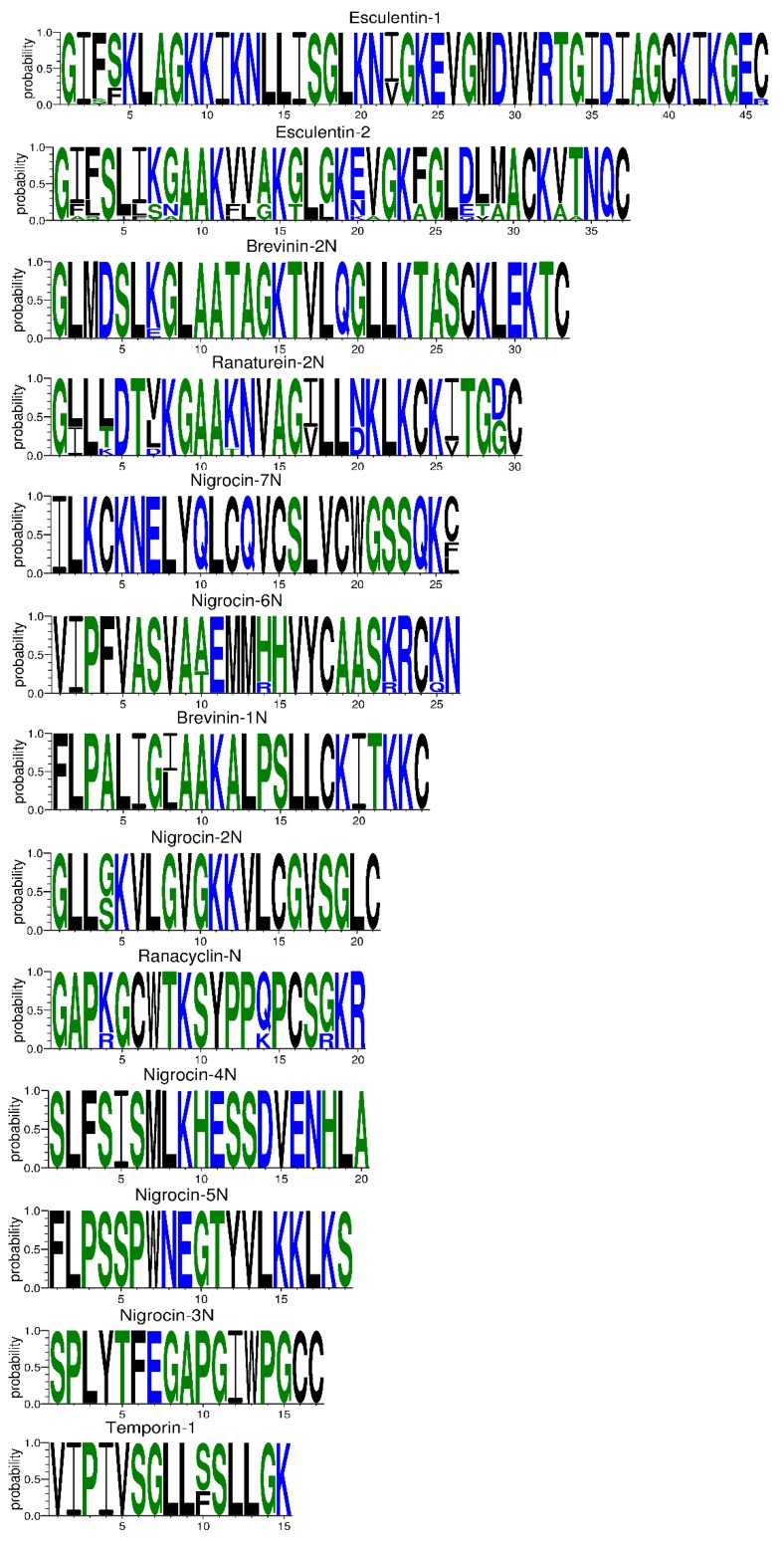
The primary structure of mature peptides of 13 antimicrobial peptide (AMP) families from *P. nigromaculatus*. The blue letters represent hydrophilic residues, including Arginine (R), Lysine (K), Aspartic acid (D), Glutamic acid (E), Asparagine (N), Glutamine (Q); the green letters represent neutral residues, namely Serine (S), Glycine (G), Histidine (H), Threonine (T), Alanine (A), and Proline (P); the black letters represent hydrophobic residues, including Tyrosine (Y), Valine (V), Leucine (L), Isoleucine (I), Phenylalanine (F), Tryptophane (W), Methionine (M), and Cysteine (C).

The 106 AMP cDNA sequences from *P. nigromaculatus* encoded 38 mature peptides of 13 families (Figure 11). All these families were rich in Leucine (L). Family nigrocin-6N was rich in hydrophobic amino acid residues Alanine (A); family nigrocin-4N and nigrocin-5N were rich in polar amino acid Serine (S). The molecular masses of these 38 AMPs ranged from 1495.8 to 4876.9, and the pI values ranged from 4.00 to 10.06. Except for the family nigrocin-3N (pI=4.00) and nigrocin-4N (pI = 5.26), the remaining 11 families were alkaline AMPs (pI = 8.02~10.06) (Figure 7). Most AMPs were hydrophobic, with families nigrocin-2N and temporin-1 displaying strong hydrophobicity, while families nigrocin-4N, nigrocin-5N, and ranacyclin-N showed hydrophilicity (Figure 8). The predicted values of instability indices for nigrocin-5N (50.12), nigrocin-6N (49.29), and ranacyclin-N (55.08) suggested poor stability, while the remaining families had extremely high stability (Figure 9). The secondary structure of mature peptides of family ranatuerin-2N, nigrocin-6N, esculentin-2, and nigrocin-4N was in all-α conformation (Table A3).

### 3.5. Relationship between Propieces and Mature Peptides

**Figure 12:**
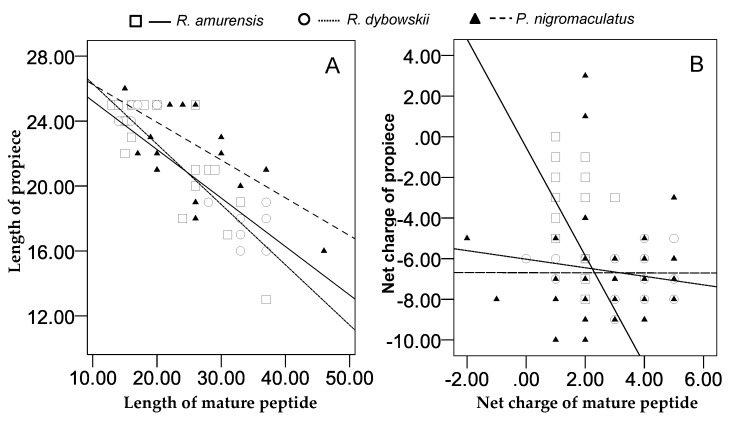
Relationship between the acidic propiece and the mature peptide of antimicrobial peptides (AMPs) identified from three anuran species. A: The linear correlation between the length (the number of amino acid residues) of the propiece and the mature peptide (*R. amurensis*: *r* = −0.645, *p* = 0.000; *R. dybowskii*: *r* = −0.931, *p* = 0.000; *P. nigromaculatus*: *r* = −0.858, *p* = 0.000); B: linear correlation between the net charges of the propiece and the mature peptide (*R. amurensis*: *r* = −0.399, *p* = 0.000; *R. dybowskii*: *r* = −0.311, *p* = 0.005; *P. nigromaculatus*: *r* = 0155, *p* = 0.113).

For all 266 cDNA sequences, the number of amino acid residues of the propiece was negatively correlated with that of the mature peptide of the same sequence (*r* = −0.762, *p* = 0.000). Similar significant correlation was recorded for cDNAs of each species (*R. amurensis*: *r* = −0.645, *p* = 0.000; *R. dybowskii*: *r* = −0.931, *p* = 0.000; and *P. nigromaculatus*: *r* = −0.858, *p* = 0.000) (Figure 12A).

The net charge numbers varied for mature peptides and propieces, with greatest variation in *P. nigromaculatus*, least in *R. dybowskii*, and middle in *R. amurensis*. For each species, we detected significant correlation of net charge numbers between propiece and mature peptide, negative correlation in *R. amurensis* (*r* = −0.399, *p* = 0.000) and *R. dybowskii* (*r* = −0.311, *p* = 0.005), and positive correlation in *P. nigromaculatus* (*r* = 0.155, *p* = 0.113) (Figure 12B). We did not detect significant correlation when data of the three species were pooled (*r* = −0.082, *p* = 0.182).

### 3.6. Pattern of Nucleotide Substitution

#### 3.6.1. t_s_/t_v_ Ratio of Propieces to Mature Peptides

The families brevinin-2AM in *R. amurensis* and nigrocin-4N in *P. nigromaculatus* each had only one sequence and were discarded from the analysis of nucleotide substitution. The *t_s_*/*t_v_* ratio of all AMP families varied greatly for all three species. The ratio of propiece of *R. amurensis* ranged from as low as 0 to as high as 6.33 with a mean of 2.06 ± 2.07; The ratio of mature peptides of this species ranged between 0.00 and 4.04, and averaged 1.70 ± 1.00; The ratio of whole AMP cDNA was 2.00 ± 1.63 on average with a range between 0.00 and 6.13. In *R. dybowskii*, the ratio was 1.05 ± 0.74 on average (0.00–1.63) for propiece, 2.70 ± 2.05 (0.78–7.31) for mature peptide, and 1.71 ± 0.53 (0.90–2.78) for the whole AMP cDNA. In *P. nigromaculatus*, the ratios of the two peptide sections were 1.51 ± 1.23 (0.23–4.73) and 3.35 ± 4.05 (0.00–12.92), respectively, and 3.08 ± 2.60 (1.70–11.32) for the whole AMP cDNA (Table 2). No significant correlation of *t_s_*/*t_v_* was detected between the propiece and the mature peptide for all three species (*p* > 0.05).

#### 3.6.2. d_N_/d_S_ Ratio of Propieces and Mature Peptides Region

The number of non-synonymous substitutions (d*_N_*) and the number of synonymous substitutions (d*_S_*) were counted for propiece, mature peptide, and whole cDNA for all families, but brevinin-2AM and nigrocin-4N had only one cDNA sequence. On the mature peptide region, the values of d*_N_* and d*_S_* were <1 (d*_N_* = 0.00~0.999 and d*_S_* = 0.00~0.89), except for family esculentin-2 (d*_S_* = 1.40). Similarly, for the acidic propiece region, d*_N_* value ranged from 0.00 to 0.61 and d*_S_* value ranged from 0.00 to 0.63, except for family amurin-6N (d*_S_* = 2.05).

**Figure 13:**
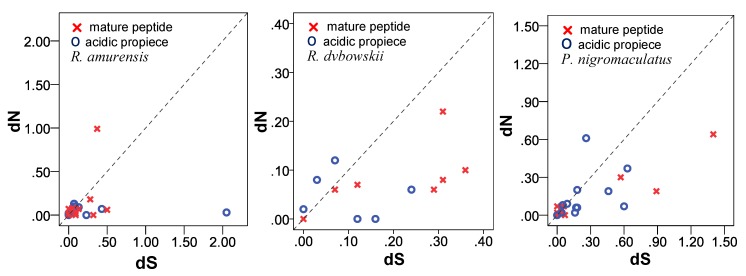
Plots of d*_N_* against d*_S_* of the propiece and the mature peptide region for three anuran species. The broken line is the neutral line (ω = 1).

To further visualize the selection mode of the propiece and mature peptide region, the d*_N_* was plotted against d*_S_* values for each family of the three species (Figure 13). The distribution of d*_N_* and d*_S_* values of *R. amurensis* in both propiece and mature peptide regions was relatively compact below 0.50, except for temporin-AM (d*_N_* = 0.99, mature peptide region) and amurin-6N (d*_S_* = 2.05, propiece region). For the mature peptide region, families amurin-3AM, amurin-5AM, ranacyclin-2AM, and temporin-AM plotted above the neutral line ω = 1, while families amurin-4AM, amurin-6AM, amurin-7AM, amurin-8AM, amurin-9AM, amurin-10AM, japonicin-1AM, palustrin-2AM, and ranalexin-1AM plotted below the neutral line ω = 1. For the acidic propiece region, families amurin-3AM, amurin-4AM, amurin-5AM, amurin-8AM, amurin-9AM, amurin-10AM, and japonicin-1AM plotted above the line ω = 1, while families amurin-6AM, amurin-7AM, palustrin-2AM, ranacyclin-2AM, ranalexin-1AM and temporin-AM plotted below the neutral line. The pattern of d*_N_* and d*_S_* values suggests that the mature peptide and acidic propiece region were all differentially selected.

The distribution of d*_N_* and d*_S_* values of *R. dybowskii* was relatively compact and <0.40 for both propiece and mature peptide regions. All data for the mature peptide region plotted below the ω = 1 line, except for the family dyowsin-3 (ω = 1), suggesting that most AMPs were under negative selection in this region. In contrast for the acidic propiece region, families dyowsin-1, dyowsin-2, dyowsin-3, and chensirin-3 plotted above the ω = 1 line, while families amurin-3, chensirin-1, and chensirin-2 plotted below the neutral line ω = 1, suggesting that this region was differentially selected.

The range of d*_N_* and d*_S_* values for *P. nigromaculatus* was relatively wide, from 0.00 to 1.40. The data of the mature peptide region for families brevinin-1N, esculentin-1, nigrocin-2N, nigrocin-6N, and ranacyclin-N plotted above the ω = 1 line, while that for families brevinin-2N, esculentin-2, nigrocin-3N, nigrocin-5N, nigrocin-7N ranaturein-2N, and temporin-1 plotted below the neutral line ω = 1. Similarity for the acidic propiece region, families esculentin-2, nigrocin-5N, nigrocin-7N, ranacyclin-N and temporin-1 plotted above the ω = 1 line, and families brevinin-1N, brevinin-2N, esculentin-1, nigrocin-2N, nigrocin-3N, nigrocin-6N and ranaturein-2N plotted below the neutral line ω = 1. This suggested that the mature peptide and acidic propiece regions were differentially selected.

## 4. Discussion

### 4.1. Interspecific Variation of antimicrobial peptide (AMP) Composition

Each AMP has an antimicrobial spectrum, and the spectra of different AMPs can overlap to form a broad, enhanced spectrum covering almost all microbial pathogens, given that the AMP system is highly diverse [38,39]. By this means, frogs may successfully protect themselves from complex environmental pathogen communities. In this study, we obtained 266 AMP cDNA sequences from the skin of *R. amurensis*, *R. dybowskii*, and *P. nigromaculatus*. The three species varied greatly in AMP composition, with 14 families in *R. amurensis*, 7 families in *R. dybowskii* and 13 families in *P. nigromaculatus* (see Table A1, Table A2 and Table A3). AMPs of each family had similar structure and physicochemical properties, while the structure and physicochemical properties varied greatly from family to family (Table A1, Table A2 and Table A3, Figure 7, Figure 8 and Figure 9). This demonstrates that the three species sympatric to northeast China use different sets of highly diverse AMPs to cope with similar microbial environments.

All three frog species exhibited two levels of AMP diversity, within-family diversity, and among-family diversity. Nucleotide variation (Figure 4) and amino acid residue variation was recorded within families. Change of amino acid residue may result in alteration of the antimicrobial spectrum and efficiency [40,41]. However, family members are often highly homologous. Therefore, the within-family variation of mature peptides may not significantly contribute to expansion of the antimicrobial spectrum. Instead, it might contribute to enhancement of antimicrobial efficiency against a specific group of pathogens with a few more alternative agents. The number of family members varied from family to family (Table A1, Table A2 and Table A3), suggesting frogs have differential antimicrobial efficiency against different pathogens instead of indiscriminate defense. This strategy allows them to reduce energy costs for weak enemies and focus on deadly pathogens.

In contrast, among-family diversity showed great divergence of structure and physicochemical properties (Figure 7, Figure 8 and Figure 9). Such divergence contributes to expansion of the antimicrobial spectrum [42,43]. For each species, all AMP families were phylogenetically related (Figure 1, Figure 2 and Figure 3), suggesting the expansion of AMP genes by duplication and diversification [44]. It was posited that such duplication is driven by positive selection where variants benefit the animal with the enhanced and expanded antimicrobial spectrum [17,22]. However, we detected only a few families that were subject to positive selection, while most families were under either near neutral or various degree of negative selection (Figure 13). Negative selection suggests that the present forms and physicochemical properties are essential to cope with present pathogenic attacks, and changes may reduce the antimicrobial spectrum and efficiency, leading to reduction of individual fitness. When the defense network made up with such essential AMPs is adequate, the importance of some other AMPs could be reduced and selection pressure against mutations could decline. Given modification by mutation that leads to addition of novel functions that improve fitness, the AMPs would shift to be under positive selection. Apparently, negative selection can be relaxed only if the number of AMP variants is large enough, enabling some of them to become functional backups of others. In this scenario, gene duplication events, i.e., addition of family members and numbers, would have more essential evolutionary significance than diversification events.

This expectation was supported by the results of our study. As stated above, these species can share common habitat but also have discrete habitat preferences in areas where their ranges overlap. The range size and habitat variability of the three species rank in increasing order as *R. dybowskii*, *R. amurensis*, and *P. nigromaculatus*. AMP diversity detected here (Table A1, Table A2 and Table A3) ranked in the same order, least in *R. dybowskii* (79 cDNA sequences encoding 24 mature peptides of 7 families), medium in *R. amurensis* (81 cDNA sequences encoding 33 mature peptides of 14 families), and greatest in *P. nigromaculatus* (106 cDNA sequences encoding 38 mature peptides of 13 families). The frogs of all three species were trapped in various habitats and a group of each species was raised in clean water. The degenerate primers for RT-PCR could theoretically amplify all families of AMPs of anurans and large numbers of clones were sequenced for each species. Therefore, we believe the transcripts of each species could represent the greatest AMP diversity although the isolation might not have exhausted all. The association between AMP diversity and habitat variability strongly implies gene duplication is the fundamental approach of adaptive evolution of the whole AMP system.

Time-calibrated phylogenetic analysis showed that *P. nigromaculatus* separated from the genus Rana about 46.57 mya, and *R. amurensis* and *R. dybowskii* substantially separated around 13.25 mya [45]. However, our phylogenetic analysis showed that diversification of AMP was not associated with this speciation process (Figure 5). AMPs of *P. nigromaculatus* did not exhibit early separation from those of the two Rana species. Instead, the same clade was shared by different species, but further diverged into species-specific families. This pattern suggests the evolution rate of AMPs of each family and among families is dynamic and strongly influenced by variation in habitat (pathogenic communities). During co-evolution between AMPs and pathogenic microorganisms [46], each may have chances to shift from being under negative selection to neutral or positive selection based upon the dynamics of pathogenic pressure. This, in turn affects the dynamics of evolution rates.

### 4.2. Correlation between Propieces and Mature Peptides

The knowledge of mammalian α-defensins showed that the anionic propiece could prevent autocytotoxicity by neutralizing the cationicity of the mature peptide [47]. Therefore, there was a negative linear relationship between the net negative charge of the propiece and the positive net charge of the mature peptide [48]. We observed a weak but significant negative linear correlation of net charges between propiece and mature peptide in *R. amurensis* (*r* = −0.399, P=0.000) and *R. dybowskii* (*r* = −0.311, *p* = 0.005), but not in *P. nigromaculatus* (*r* = 0.155, *p* = 0.113). Our results also showed correlation to be associated with diversity of AMPs as the ranges of net charges varied greatly for the two segments in all three species. These results suggest that the “Net Charge Balance Hypothesis” is generally supported, but the requirements to prevent AMPs’ autocytotoxicity by charge neutralization are not strict. This greatly relaxes natural selection against amino acid substitutions, highlighting an important mechanism for retention of high diversity of both mature peptide and propiece.

Interestingly, we found a strong negative linear correlation between the length of propiece and the length of mature peptide for the 266 AMP genes (*r* = −0.762, *p* = 0.000), and the same relationship was observed in each species (Figure 12A). This could not be explained with the Net Charge Balance Hypothesis, because the number of amino acid residues is not necessarily associated with net charges. Instead, we expect that the cut point between propiece and mature peptide for maturation (often KR, ER, and RR) may be occasionally cancelled by mutation and occasionally appear again at another position due to mutation, but the new mature peptide and propiece cropped by this means still maintain AMP characteristics and functions. We did not go deep into the Cut Point Sliding Hypothesis in the present study but it should be further tested in future studies.

## 5. Conclusions

The three partially sympatric anuran species were characterized by high diversity of AMPs. All AMPs were phylogenetically related but not associated with speciation. Diversification driving addition of favored functions and efficiency depends on the expansion of family members and numbers. Cut Point Sliding might be a new mechanism of diversification.

## Figures and Tables

**Table 1:** Primer sequences used for antimicrobial peptides’ (AMPs’) amplification.

Primer	Sequence (5′–3′)	Ta
PS1	GGGAC(C/A) ATCAACTTATCTTGT	54 °C
PS2	GGGACCATCTCCTT(A/G)TCTCTCTGT	54 °C
PS3	GGGAT(G/C)ATCTCCTTA(A/T)CTCTCTGT	56 °C
PS4	GGAACCATCTC(A/G)TCTCTCTGT	55 °C
PS5	GGGACCATCAACTTCTCTCTCTGT	57 °C
PS6	GGGACCATCAACTT(A/T)TCT(C/T)T(C/T/A)TGT	55 °C
PS7	GGGACCATCTCCTTATC(T/G)CT(C/T)TG	54 °C
PS8	GGGACCATCA(G/C)CTTACT(C/T)TGT	56 °C
PS9	GGGACCATC(G/T)CCCT(T/A)TCTCTCTG	55 °C
PS10	GGG(A/G)TC(A/G)TCTC(G/C)TTATCTCTCTGT	55 °C
PC	TTTTTTTTTTTTTTTTTTTTTTT(C/G/A)	50 °C

**Table 2:** The *t_s_*/*t_v_* ratios of propiece and mature peptide of antimicrobial peptides (AMPs)AMPs in three anuran species.

Species	AMP family	ts/tv		
		Propiece	Mature Peptide	Whole AMP *
*R. amurensis*	Amurin-3AM	5.31	2.60	2.43
	Amurin-4AM	1.91	1.57	1.43
	Amurin-5AM	-	-	6.13
	Amurin-6AM	0.08	1.28	1.37
	Amurin-7AM	0.69	2.04	0.63
	Amurin-8AM	2.27	-	1.62
	Amurin-9AM	0.00	1.99	0.00
	Amurin-10AM	-	1.32	2.16
	Japonicin-1AM	-	2.00	2.72
	Palustrin-2AM	6.33	1.01	1.76
	Ranacyclin-2AM	2.00	0.00	0.00
	Ranalexin-1AM	1.91	0.83	4.39
	Temporin-AM	0.09	4.04	1.32
	Brevinin-AM	/	/	/
*R. dybowskii*	Amurin-3	1.52	0.92	1.58
	Chensirin-1	-	1.88	1.83
	Chensirin-2	-	7.31	0.90
	Chensirin-3	1.63	0.78	1.38
	Dybowsin-1	0.00	2.71	2.78
	Dybowsin-2	-	3.29	1.66
	Dybowsin-3	-	2.00	3.20
*P. nigromaculatus*	Brevinin-1N	2.00	0.00	1.77
	Brevinin-2N	0.23	-	3.56
	Esculentin-1	1.39	-	1.70
	Esculentin-2	1.17	2.08	11.32
	Nigrocin-2N	-	-	1.72
	Nigrocin-3N	0.91	-	2.36
	Nigrocin-5N	4.73	0.00	1.38
	Nigrocin-6N	0.69	5.83	1.74
	Nigrocin-7N	0.98	3.56	3.57
	Ranacyclin-N	-	1.18	1.63
	Ranaturein-2N	0.68	1.22	3.04
	Temporin-1	2.36	12.92	2.43
	Nigrocin-4N	/	/	/

- indicates tv was 0 and the ratio was absent; * whole AMPs here included partial signal peptide, whole propiece, and whole mature peptide; / indicates the ratio could not be calculated because the family had only one cDNA sequence.

## Appendix A

**Table A1:** The physicochemical and structural properties of mature peptides from *R. amurensis*.

NO.	AF	MW	pI	Mature Peptides	II	GRAVY	SS
1	Ranacyclin-2AM	3823.50	10.31	LAKALGANKVLGQIVRLANSAARDTTQNVLSAITCKI	0.85	0.38	all-α
				CHHHHCCHHHHHHHHHHHHHHHHHHHHHHHHHHHCCC			
		3857.50	10.31	LAKALGANKVLGQIVRFANSAARDTTQNVLSAITCKI	0.85	0.35	all-α
				CHHHHCCHHHHHHHHHHHHHHHHHHHHHHHHHHHCCC			
2	Brevinin-2AM	3351.00	9.24	GLMDSLKGLAATAGKTVLQGLLKTASCKLEKTC	22.43	0.27	all-α
				CCHHHHHHHHHHHHHHHHHHHHHHHHHHCCCCC			
3	Palustrin-2AM	3367.00	9.51	GIWDSIKTFGKKFALNIMDKIKCKIGGGCPP	35.26	−0.03	all-α
				CHHHHHHHHCHHHHHHHHHHHHCCCCCCCCC			
4	Amurin-6AM	2872.50	9.90	SLLGLLKGAGKTLLSAGLNKIACKLTGKC	9.55	0.60	all-α
				CHHHHHHHHHHHHHHHCCHHHHHHHCCCC			
		3007.70	9.51	GLMDFLKGAGKKLLAAGLDKLNCKLTGKC	−23.21	0.18	all-α
				CCCHHHHHHCHHHHHHCCCCCCEEECCCC			
5	Amurin-5AM	3114.60	8.71	ALPFLPSLDADIVQQVCCLPRRRCTNGK	85.13	0.01	mixed
				CCCCCCCCCCHHHHHHHHCCCCCCCCCC			
		3015.50	8.00	ALPFLPSLDADIVQQVCCLPRGRCTNGK	41.19	0.16	mixed
				CCCCCCCCCCHHHHHHHHCCCCCCCCCC			
		3015.50	8.00	ALPFLPSLDADIVQQVCCLPRRGCTNGK	61.64	0.16	mixed
				CCCCCCCCCCHHHHHHHHCCCCCCCCCC			
		3104.60	8.71	ALPFLPSLDADIVQQVCCLSRRRCTNGK	87.83	0.04	mixed
				CCCCCCCCCCHHHHHHHHCCCCCCCCCC			
		3130.70	8.71	ALPFLPSLDADIVQQVCCLLRRRCTNGK	87.83	0.21	all-α
				CCCCCCCCCHHHHHHHHHHHHCCCCCCC			
		3056.60	9.22	ALPFLPSLGADIVQQVCCLPRRRCTNGK	82.10	0.13	mixed
				CCCCCCCCCCHHHHHHHHCCCCCCCCCC			
6	Amurin-3AM	2947.50	8.70	FLPGLKRCCFEVMSSVFCAITRRPDA	77.14	0.45	mixed
				CCCCCHHHHHHHHHHHHHEECCCCCC			
		2919.50	8.68	FLPGLKRCCFEVMSSVFCAITRKPDA	44.80	0.47	mixed
				CCCCHHHHHHHHHHHHHHEECCCCCC			
		2816.40	7.95	FLPGLKGCCFEVMSSVFCAIPRKPDA	24.99	0.59	mixed
				CCCCCCHHHHHHHHHHHHEECCCCCC			
		2788.30	7.94	FLPGLKGCCFEVMSSVFCAIPKKPDA	27.89	0.62	mixed
				CCCCCCHHHHHHHHHHHHECCCCCCC			
7	Amurin-8AM	2828.50	9.51	GFVGKYMGKISADHLKMIICKVTGKC	6.40	0.33	mixed
				CCCHHHCCCCCHHHHHHHHHEECCCC			
8	Amurin-4AM	2621.10	8.59	EAKINFAEMFAAVKKILQQGVPMG	29.56	0.26	all-α
				CCCCCHHHHHHHHHHHHHHCCCCC			
		2646.10	9.70	EAKINFAEMFAAVKKILQQGVPRG	26.03	-0.01	all-α
				CCCCCHHHHHHHHHHHHHHCCCCC			
		2603.10	8.59	EAKINFAEIFAAVKKILQQGVPMG	37.59	0.37	all-α
				CCCCCHHHHHHHHHHHHHHCCCCC			
		2611.10	10.00	EAKINFAKIFAAVKKILHQGVPMG	4.43	0.36	all-α
				CCCCCHHHHHHHHHHHHHHCCCCC			
9	Ranalexin-1AM	2286.90	8.90	FFPLLLSALPSFLCLVFKKC	40.51	1.62	all-α
				CCHHHHHHHHHHHHHHHHCC			
		2252.80	8.90	FLPLLLSALPSFLCLVFKKC	40.51	1.67	all-α
				CCHHHHHHHHHHHHHHHHCC			
10	Amurin-7AM	2456.80	12.52	SLGRFQGRFGRRTHRKHFVN	32.95	-1.31	mixed
				CCCCCCCCCCCCCHHHHHCC			
11	Japonicin-1AMa	2025.50	9.39	FLPPTLVGLAFCKLFKKC	13.06	1.03	all-α
				CCCCHHHHHHHHHHHHHC			
		1517.90	9.39	FLGLAFCKLFKKC	−8.86	1.12	all-α
				CCCCHHHHHHHHC			
12	Amurin-9AM	1745.10	9.39	FLPFFAACAITKKCGK	55.61	0.79	all-α
				CCCHHHHHHHHHHHCC			
13	Temporin –Ama	1705.10	8.75	ILPLLGNLLNDLLRK	42.19	0.68	all-α
				CCCHHHHHHHHHHHC			
		1639.10	11.00	FLPVLISLIGRLLGK	29.35	1.56	all-α
				CCHHHHHHHHHHHCC			
		1487.80	8.75	FVSLLTNLLGLLGK	−7.51	1.44	all-α
				CHHHHHHHHHHHCC			
14	Amurin-10AM	1537.80	9.75	FLGSILGSLASLFRG	16.51	1.27	all-α
				CCHHHHHHHHHHHCC			
		1640.90	12.00	FLGSILGSRAFLFRG	29.35	0.95	mixed
				CCCHHHCCCEEEECC			
		1597.90	9.75	FLGSILGSLAFLFRG	16.51	1.51	all-α
				CCHHHHHHHHHHHCC			

AF (AMP Family); MW (Molecular Weight); The upper line is deduced amino acid sequence of mature peptide, and the lower line is the computed secondary structure wherein C stands for random coil, E stands for extended strand, H stands for helix [35]; II (Instability index) provides an estimate of the stability of the protein in a test tube, stable when II is below 40 and instable if II is above 40 [49]; GRAVY (Grand average of hydropathy) value is calculated by adding the hydropathy value for each residue and dividing by the length of the sequence, with a positive value indicating hydrophobicity and negative value indicating hydrophilicity [50]. SS (Secondary structure), sorted as all-α when H% >45% and E% <5%, all-β when H%<5% and E% >45%, α-β when H% >30% and E% >20% and mixed for all other situations [36].

**Table A2:** The physicochemical and structural properties of mature peptides from *R. dybowskii*.

NO.	AF	MW	pI	Mature Peptides	II	GRAVY	SS
1	Chensirin-3	3732.46	9.51	GLFSEVKGVLKGVGKNVAKNVAGSLLEQLKCKISGGC	6.64	0.27	all-α
				CCHHHHHHHHHHHCHHHHHHHHHHHHHHHHCHCCCCC			
		3674.42	9.79	GLFSVVKGVLKAAGKNVAKNVGGSLLEQLKCKISGGC	7.06	0.41	all-α
				CCHHHHHHHHHHHCHHHHHHHHHHHHHHHHCHCCCCC			
		3702.48	9.79	GLFSVVKGVLKGVGKNVAKNVAGSLLEQLKCKISGGC	1.43	0.47	all-α
				CCHHHHHHHHHHHCHHHHHHHHHHHHHHHHCHCCCCC			
		3702.48	9.79	GLFSVVKGVLKAVGKNVAKNVGGSLLEQLKCKISGGC	4.76	0.47	all-α
				CCHHHHHHHHHHHCHHHHHHHHHHHHHHHHCHCCCCC			
		3289.99	9.60	GLFSVVKGVLKGVGKNVAGSLLEQLKCKISGGC	0.39	0.57	all-α
				CCCHHHHHHHHHHHHHHHHHHHHHHHCHCCCCC			
		3319.97	9.24	GLFSVVKGVLKGEGKNVAGSLLEQLKCKISGGC	0.68	0.34	all-α
				CCCCHHHHHHHHCCHHHHHHHHHHHHCHCCCCC			
		3213.89	9.60	GILSAVKGVLKGVGKNVAGSLLDQLKCKLSGGC	−2.18	0.53	all-α
				CCHHHHHHHHHHHHHHHHHHHHHHHHHHHCCCC			
		3289.99	9.60	GLLSVFKGVLKGVGKNVAGSLLDQLKCKISGGC	−9.13	0.56	all-α
				CCHHHHHHHHHHHHHHHHHHHHHHHHCHECCCC			
		3275.96	9.60	GLFSVVKGVLKGVGKNVAGSLLDQLKCKISGGC	−5.45	0.57	all-α
				CCCHHHHHHHHHHHHHHHHHHHHHHHCHECCCC			
2	Dybowsin-1	2908.64	10.07	GLMGILKVAVNKLLAAGMNKPRCKAAHC	12.89	0.46	all-α
				CCCCHHHHHHHHHHHHCCCCHHHCCCCC			
		3000.69	9.70	GLMDIFKVAVNKLLAAGMNKPRCKAAHC	6.71	0.31	all-α
				CCCCHHHHHHHHHHHHCCCCHHHCCCCC			
		2966.68	9.70	GLMDILKVAVNKLLAAGMNKPRCKAAHC	15.92	0.35	all-α
				CCCHHHHHHHHHHHHHCCCCHHHCCCCC			
3	Dybowsin-2	2361.93	9.39	FFSLAFAGFPKFLCFLFKKC	11.62	1.26	mixed
				CCCCCCCCCCHHHHHHHHHC			
		2312.00	9.39	FLPLFFAALPKFLCLVLKKC	34.15	1.60	all-α
				CCHHHHHHHHHHHHHHHHCC			
		2235.82	9.39	FLSLALAGFSKFLCLVFKKC	11.62	1.47	all-α
				CCCHHHHHHHHHHHHHHHCC			
		2269.83	9.39	FFSLALAGFSKFLCLVFKKC	11.62	1.42	all-α
				CCCHHHHHCHHHHHHHHHCC			
4	Dybowsin-3	1910.42	8.96	FLIGMTQGLICLITRKC	14.61	1.21	mixed
				CCCCCCHHHHHHHECCC			
5	Chensirin-1	1611.94	5.81	VFPLVGNLLNDLLGK	5.19	0.89	all-α
				CCCCHHHHHHHHHCC			
		1577.93	5.81	VLPLVGNLLNDLLGK	5.19	0.96	all-α
				CCCCHHHHHHHHHCC			
6	Amurin-3.	1487.93	8.75	ILPILAPLIGGLLGK	63.26	1.73	all-α
				CCCHHHHHHHHHCCC			
		1406.82	8.75	ILPILSLIGGLLGK	53.31	1.79	all-α
				CCHHHHHHHHHCCC			
7	Chensirin-2	1450.78	9.70	IIPLSLGYFAKKT	0.48	0.66	mixed
				CCCCCHHHCECCC			
		1476.82	9.53	IIPLPLGYYAKKT	43.01	0.29	mixed
				CCCCCCCCCCCCC			
		1460.82	9.70	IIPLPLGYFAKKT	15.30	0.60	mixed
				CCCCCCCCCCCCC			

AF (AMP Family); MW (Molecular Weight); The upper line is deduced amino acid sequence of mature peptide, and the lower line is the computed secondary structure wherein C stands for random coil, E stands for extended strand, H stands for helix [35]; II (Instability index) provides an estimate of the stability of the protein in a test tube, stable when II is below 40 and instable if II is above 40 [49]; GRAVY (Grand average of hydropathy) value is calculated by adding the hydropathy value for each residue and dividing by the length of the sequence, with a positive value indicating hydrophobicity and negative value indicating hydrophilicity [50]. SS (Secondary structure), sorted as all-α when H% >45% and E% <5%, all-β when H%<5% and E% >45%, α-β when H% >30% and E% >20% and mixed for all other situations [36].

**Table A3:** The physicochemical and structural properties of mature peptides from *P. nigromaculatus*.

NO.	AF	MW	pI	Mature Peptides	II	GRAVY	SS
1	Esculentin-1	4816.84	9.63	GIFSKLAGKKIKNLLISGLKNIGKEVGMDVVRTGIDIAGCKIKGEC	3.25	0.23	mixed
				CCCCCCCCCHHHHHHHHHHHHHHHHHHHHHHHHCCCECCEEECCCC			
		4876.94	9.63	GIFFKLAGKKIKNLLISGLKNIGKEVGMDVVRTGIDIAGCKIKGEC	−0.01	0.30	mixed
				CCCCCCCCCHHHHHHHHHHHHHHHHHHHHHHHHCCCCCCEEECCCC			
		4756.75	9.63	GISSKLAGKKIKNLLISGLKNIGKEVGMDVVRTGIDIAGCKIKGEC	7.44	0.15	mixed
				CCCCCCCCHHHHHHHHHHHHHHHHHHCHHHHHHCCCECCEEECCCC			
		4802.82	9.63	GIFSKLAGKKIKNLLISGLKNVGKEVGMDVVRTGIDIAGCKIKGEC	−8.14	0.22	mixed
				CCCCCCCCCHHHHHHHHHHHHHHHHHHHHHHHHCCCECCEEECCCC			
		4869.89	10.02	GIFSKLAGKKIKNLLISGLKNIGKEVGMDVVRTGIDIAGCKIKGER	−6.30	0.07	mixed
				CCCCCCCCCHHHHHHHHHHHHHHHHHCHHHHHHCCCECCEEEECCC			
2	Esculentin-2	3782.47	9.60	GFLSLLSNAAKFLGKTLLKNVGKAGLETAACKATNQC	9.60	0.27	all-α
				CHHHHHHHHHHHHHHHHHHHHHHHHHHHHHHHHCCCC			
		3794.68	9.93	GIFSLIKGAAKVVAKGLGKKVGKFGLDLMACKVTNQC	−10.73	0.51	all-α
				CCCHHHHHHHHHHHHHHHHHHHHHHHHHHHEECCCCC			
		3795.62	9.51	GIFSLIKGAAKVVAKGLGKEVGKFGLDLMACKVTNQC	−8.44	0.52	all-α
				CCCHHHHHHHHHHHHHHHHHHHHHHHHHHHHHCCCCC			
		3735.52	9.51	GISSLIKGAAKVVAKGLGKEVGKFGLDLMACKVTNQC	−3.23	0.43	all-α
				CCCHHHHHHHHHHHHHHHHHHHHHHHHHHHHHHCCCC			
		3602.31	9.90	GALSIFSAAAKLLGKTLLKNAGKAGLQVAACKAANQC	12.83	0.54	all-α
				CHHHHHHHHHHHHHHHHHHHHHHHHHHHHHHHHHCCC			
3	Brevinin-2N	3351.98	8.02	GLMDSLEGLAATAGKTVLQGLLKTASCKLEKTC	27.57	0.28	all-α
				CCHHHHHHHHHHHHHHHHHHHHHHHHCCCCCCC			
		3351.04	9.24	GLMDSLKGLAATAGKTVLQGLLKTASCKLEKTC	22.43	0.27	all-α
				CCHHHHHHHHHHHHHHHHHHHHHHHHHHCCCCC			
	Ranaturein-2N	3001.64	9.24	GILTDTLKGAAKNVAGVLLDKLKCKITGGC	−10.28	0.42	all-α
				CCCHHHHHHHHHHHHHHHHHHCCCEECCCC			
		3001.64	9.24	GILKDTLKGAATNVAGVLLDKLKCKITGGC	−18.12	0.42	all-α
				CCCHHHHHHHHHHHHHHHHHHHCCEECCCC			
		3012.71	9.60	GILLNTLKGAAKNVAGVLLDKLKCKITGGC	−8.10	0.57	all-α
				CCHHHHHHHHHHHHHHHHHHHCCCEECCCC			
		3070.74	9.24	GLLLDTVKGAAKNVAGILLNKLKCKITGDC	−1.80	0.47	all-α
				CCHHHHHHHHHHHHHHHHHHHCCCEECCCC			
		3086.70	8.79	GLLLDTDKGAAKNVAGILLNKLKCKITGDC	−3.67	0.21	all-α
				CCCCHHHHHHHHHHHHHHHHHCCCEECCCC			
		3056.72	9.24	GLLLDTVKGAAKNVAGILLNKLKCKVTGDC	−4.63	0.46	all-α
				CCHHHHHHHHHHHHHHHHHHHCCCEECCCC			
5	Nigrocin-6N	2893.45	8.86	VIPFVASVATEMMHHVYCAASKRCKN	53.88	0.35	all-α
				CCCHHHHHHHHHHHHHHHHHHHHCCC			
		2921.42	8.05	VIPFVASVATEMMHHVYCAASRRCQN	67.88	0.35	all-α
				CCCHHHHHHHHHHHHHHHHHHHHHCC			
		2882.47	9.39	VIPFVASVAAEMMRHVYCAASKRCKN	28.95	0.40	all-α
				CCCHHHHHHHHHHHHHHHHHHHHCCC			
		2863.42	8.86	VIPFVASVAAEMMHHVYCAASKRCKN	46.48	0.45	all-α
				CCCHHHHHHHHHHHHHHHHHHHHCCC			
6	Nigrocin-7N	2987.60	8.50	ILKCKNELYQLCQVCSLVCWGSSQKL	24.63	0.30	mixed
				CCCCHHHHHHHHHHHHEEEECCCCCC			
		2977.58	8.40	ILKCKNELYQLCQVCSLVCWGSSQKC	27.89	0.25	mixed
				CCCCHHHHHHHHHHHHEEEECCCCCC			
		3021.62	8.50	ILKCKNELYQLCQVCSLVCWGSSQKF	27.89	0.26	mixed
				CCCCHHHHHHHHHHHHEEEECCCCCC			
7	Brevinin-1N	2513.22	9.70	FLPALIGIAAKALPSLLCKITKKC	34.61	1.12	all-α
				CCCHHHHHHHHHHHHHHHHHHHCC			
		2513.22	9.70	FLPALIGLAAKALPSLLCKITKKC	38.15	1.09	all-α
				CCCHHHHHHHHHHHHHHHHHHHCC			
8	Nigrocin-2N	2030.56	9.39	GLLSKVLGVGKKVLCGVSGLC	−6.65	1.21	all-α
				CCCHHHHCCCCEEEECCCCCC			
		2000.53	9.39	GLLGKVLGVGKKVLCGVSGLC	−10.69	1.23	all-α
				CCCCHHHCCCCEEEECCCCCC			
9	Nigrocin-4N	2244.50	5.26	SLFSISMLKHESSDVENHLA	24.52	−0.09	all-α
				CCCCEEECCCCCCHHHHHHC			
10	Ranacyclin-N	2148.49	9.70	GAPKGCWTKSYPPQPCSGKR	58.31	−1.25	mixed
				CCCCCCCCCCCCCCCCCCCC			
		2148.53	9.90	GAPKGCWTKSYPPKPCSGKR	35.28	−1.27	mixed
				CCCCCCCCCCCCCCCCCCCC			
		2247.62	10.06	GAPKGCWTKSYPPQPCSRKR	72.19	−1.46	mixed
				CCCCCCCCCCCCCCCCCCCC			
		2176.50	9.85	GAPRGCWTKSYPPQPCSGKR	54.54	−1.28	mixed
				CCCCCCCCCCCCCCCCCCCC			
11	Nigrocin-5N	2194.56	9.53	FLPSSPWNEGTYVLKKLKS	50.12	−0.48	mixed
				CCCCCCCCCCHHHHHHCCC			
12	Nigrocin-3N	1798.06	4.00	SPLYTFEGAPGIWPGCC	38.86	0.28	mixed
				CCCCEECCCCCCCCCCC			
13	Temporin-1	1555.96	8.72	VIPIVSGLLFSLLGK	−3.91	1.83	all-α
				CCCHHHHHHHHHHCC			
		1495.87	8.72	VIPIVSGLLSSLLGK	8.93	1.59	all-α
				CCCHHHHHHHHHHCC			

AF (AMP Family); MW (Molecular Weight); The upper line is deduced amino acid sequence of mature peptide, and the lower line is the computed secondary structure wherein C stands for random coil, E stands for extended strand, H stands for helix [35]; II (Instability index) provides an estimate of the stability of the protein in a test tube, stable when II is below 40 and instable if II is above 40 [49]; GRAVY (Grand average of hydropathy) value is calculated by adding the hydropathy value for each residue and dividing by the length of the sequence, with a positive value indicating hydrophobicity and negative value indicating hydrophilicity [50]. SS (Secondary structure), sorted as all-α when H% >45% and E% <5%, all-β when H%<5% and E% >45%, α-β when H% >30% and E% >20% and mixed for all other situations [36].
